# Bayes at the Bedside: Biomarkers in Situations of Clinical Uncertainty

**DOI:** 10.3390/diagnostics16111699

**Published:** 2026-05-31

**Authors:** Uwe Klaus Zettl, Michael Hecker

**Affiliations:** Neuroimmunology Section, Department of Neurology, Rostock University Medical Centre, 18147 Rostock, Germany

**Keywords:** multiple sclerosis, neurofilament light chain, biomarkers, Bayesian inference, diagnostic uncertainty, disease activity

## Abstract

Laboratory biomarkers influence a large proportion of clinical decision-making, yet their application is often limited by incomplete validation and context-dependent interpretability. Serum neurofilament light chain (sNfL), a biomarker of neuroaxonal injury in multiple sclerosis (MS), exemplifies this challenge. Although associated with inflammatory activity, lesion burden, and disability progression at the population level, its translation into individual patient management remains problematic. In this Perspective, we synthesise current literature on sNfL in MS and apply Bayesian diagnostic reasoning as a conceptual framework for its interpretation in individualised MS care. The need for such a framework arises from the heterogeneity of MS pathology, in which subclinical inflammation and neurodegeneration may occur as partly dissociated processes that are incompletely captured by clinical and radiological measures. Consequently, substantial uncertainty persists in disease monitoring and therapeutic decision-making. In this setting, sNfL may provide complementary information, but its interpretation is complicated by biological variability, methodological differences, confounding factors (e.g., age, body mass index, and comorbidities), and the absence of universally validated thresholds. We argue that sNfL should be interpreted within a Bayesian framework, in which biomarker results modify rather than determine the probability of disease activity. Its clinical utility is likely greatest when the pre-test probability is intermediate but remains constrained by uncertainty in both test characteristics and clinical context, leading to uncertainty propagation. Overall, sNfL should be interpreted longitudinally and within multimodal clinical decision models. Further prospective studies are needed to better define its role in individualised MS management.

## 1. Introduction

Laboratory investigations are an integral component of modern medicine and are estimated to influence 60–70% of clinical decisions [1]. In situations of diagnostic uncertainty, clinical expectations are frequently directed toward novel biomarkers that appear capable of resolving existing uncertainties [2,3].

However, such biomarkers are often still in phases of incomplete clinical validation, and their individual prognostic value or utility for therapeutic management in a given patient therefore remains insufficiently established [4,5,6]. This creates a tension between scientific innovation and clinical decision-making responsibility. In practice, clinicians are thus positioned between the need for objective evidence provided by innovative biomarkers and the recognition that their limited validation and interpretative ambiguity may introduce additional uncertainty into the decision-making process [7,8].

A comparable dynamic is currently emerging in routine clinical care in the context of the biomarker serum neurofilament light chain (sNfL) in patients with multiple sclerosis (MS) [9,10]. Against this background, we aim to discuss key epistemological considerations and to examine, from an analytical perspective, the following question: Can uncertainty resolve uncertainty?

## 2. Multiple Sclerosis

MS is a chronic immune-mediated disease of the central nervous system characterised by a heterogeneous pathological profile, including demyelination, axonal and synaptic loss, as well as neurodegenerative processes with accompanying astrogliosis [11,12,13]. Despite substantial advances in diagnostics and therapy, precise determination of disease activity in routine clinical practice remains a major challenge [14,15]. In particular, differentiating between clinical and subclinical disease activity, as well as assessing the impact of potential comorbidities on the overall clinical picture, is associated with considerable uncertainty in disease monitoring [16,17,18,19].

Traditionally, MS disease activity has been defined by clinical relapses and the accumulation of neurological disability, commonly measured using the Expanded Disability Status Scale (EDSS) [20,21]. However, with the widespread availability of magnetic resonance imaging (MRI), it has become evident that inflammatory activity frequently occurs subclinically.

On the one hand, new or contrast-enhancing lesions may arise in the absence of clinical symptoms, leading to a discrepancy between clinical manifestation and radiological activity [22,23]. This phenomenon is addressed by the concept of “no evidence of disease activity” (NEDA), which combines clinical and radiological parameters but still exhibits substantial limitations [24,25].

On the other hand, recent studies indicate that a substantial proportion of acute clinical disease activity is not necessarily associated with detectable MRI changes (26.1–43.0%) [26,27].

The growing recognition of progression independent of relapse activity (PIRA) and progression independent of relapse and MRI activity (PIRMA) further complicates the assessment of disease dynamics. Disability accumulation may occur in the absence of clinically overt relapses or new MRI lesions, suggesting neurodegenerative mechanisms beyond acute inflammatory activity [28,29,30]. These findings challenge the traditional relapse- and focal inflammation-centred model of MS and highlight that clinical stability does not necessarily equate to biological inactivity [31,32].

Taken together, these factors result in substantial clinical uncertainty regarding the true level of individual disease activity, which in turn complicates therapeutic decision-making, particularly with respect to escalation, de-escalation, or switching of disease-modifying therapy [33,34]. The resulting demand for mechanistically grounded biomarkers—along with the high expectations attached to them and external commercial influences—creates both significant opportunities and substantial risks in routine clinical practice [35,36,37].

In such clinically uncertain situations, however, the use of a test with uncertain or context-dependent interpretability itself introduces a further layer of uncertainty into clinical decision-making. This issue can currently be illustrated by the individual interpretation of sNfL levels in patients with MS in clinical practice. Here, multiple layers of uncertainty converge. These include physician uncertainty regarding clinical and subclinical disease activity, potential confounding by comorbidities, biological variability of the biomarker, and methodological uncertainties related to its validation and interpretation at the individual level.

## 3. NfL in Clinical Practice

**Figure 1:**
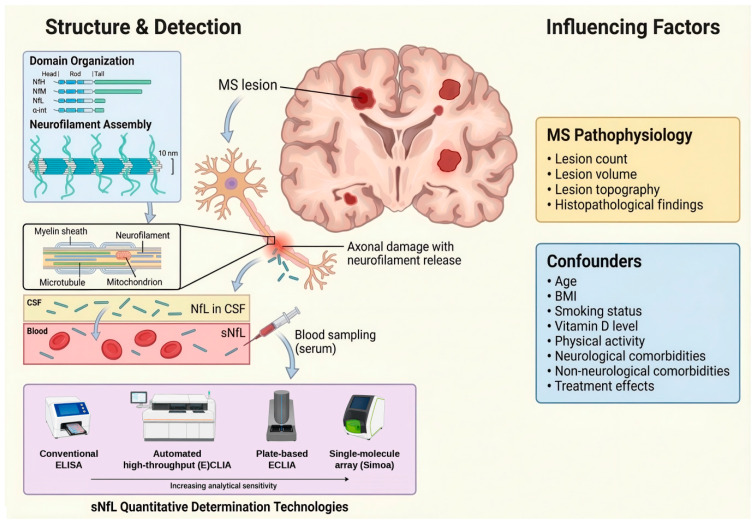
Serum NfL as a biomarker of neuroaxonal injury in MS. Schematic overview of the biological origin, detection, and interpretation of sNfL in MS. Neurofilaments, key structural components of axons, are released into the extracellular space following neuroaxonal damage associated with MS lesions. These proteins enter the CSF and subsequently the bloodstream, where sNfL can be quantified using various assay platforms that differ in analytical sensitivity. The figure also highlights key factors influencing sNfL levels, including disease-related features (e.g., lesion burden and topology) and non-specific confounders such as age, body composition, comorbid conditions, and treatment effects, which may affect the specificity of sNfL as a biomarker of disease activity. BMI, body mass index; CSF, cerebrospinal fluid; ECLIA, electrochemiluminescence immunoassay; ELISA, enzyme-linked immunosorbent assay; MS, multiple sclerosis; sNfL, serum neurofilament light chain.

Neurofilaments are structural axonal proteins that have been studied for over 70 years and are released into the cerebrospinal fluid and blood following neuroaxonal injury [38,39,40] (Figure 1). Numerous studies in patients with MS have shown that elevated sNfL levels correlate with inflammatory activity, MRI lesion burden, and future disability progression [41,42]. However, the translation of population-level associations to the individual patient remains epistemically problematic [9]. Notably, a recent study showed that in 46 of 65 patients with MS (≈71%) who had gadolinium-enhancing MRI lesions, no increase in sNfL above the 95th percentile threshold was observed [43], underscoring the limited sensitivity of sNfL for detecting radiological disease activity.

To date, no universally applicable, well-validated threshold values for sNfL have been established. Measured levels are strongly influenced by age and body mass index (BMI), are highly method-dependent (e.g., assay-specific variability), and may be substantially affected by a wide range of comorbidities, including infections, vascular events, neuropathies, or trauma, as well as by metabolic factors such as diabetes, vitamin D status, lipid parameters, or changes in blood volume [39,41,44,45,46,47].

## 4. Bayesian Decision Logic

In situations of clinical uncertainty, the application of Bayesian decision logic is both appropriate and necessary, as test results do not provide absolute truths but instead revise probabilities [48,49]. In routine clinical practice, the key question is therefore not whether an elevated sNfL level proves that MS is currently active but rather to what extent such a finding changes the probability that inflammatory disease activity is present.

### 4.1. Conceptual Starting Point

Serum NfL does not establish inflammatory disease activity in MS on its own. Rather, the measured biomarker value modifies the pre-test probability that inflammatory disease activity is present.

This Bayesian relationship can be expressed conceptually as

$$\mathrm{Posterior}\;\mathrm{probability}=\frac{\mathrm{Prior}\;\mathrm{probability}\times \mathrm{Likelihood}}{\mathrm{Evidence}}$$

In the context of sNfL interpretation:

- **Prior probability** reflects the estimated likelihood of inflammatory MS activity before biomarker testing, e.g., based on clinical symptoms and MRI findings.
- **Likelihood** describes how compatible an elevated sNfL value is with true inflammatory disease activity (i.e., how often it is observed in active vs. stable disease).
- **Evidence** represents the overall probability of observing such an sNfL value in the patient population, regardless of whether inflammatory activity is present.
- **Posterior probability** is the updated probability of inflammatory disease activity after considering the sNfL test result.

### 4.2. Classical Probabilistic Formulation

In formal notation, Bayes’ theorem can be written as

$$P(\mathrm{MS}\;\mathrm{activity}|\mathrm{sNfL}\;\mathrm{result})=\frac{P(\mathrm{sNfL}\;\mathrm{result}|\mathrm{MS}\;\mathrm{activity})\times P(\mathrm{MS}\;\mathrm{activity})}{P(\mathrm{sNfL}\;\mathrm{result})}$$

This formulation highlights that the post-test probability of inflammatory disease activity depends on the pre-test probability and on how likely the observed sNfL value is under conditions of active vs. inactive disease.

### 4.3. Transformation into Odds Form

For practical clinical reasoning, Bayes’ theorem can be reformulated using odds, which allows a more intuitive representation of probability updating.

Pre-test odds of inflammatory disease activity are defined as

$$\mathrm{Pre}\text{-}\mathrm{test}\;\mathrm{odds}\;=\;\frac{P\left(\mathrm{MS}\;\mathrm{activity}\right)}{1-P\left(\mathrm{MS}\;\mathrm{activity}\right)}$$

Post-test odds are defined analogously:

$$\mathrm{Post}\text{-}\mathrm{test}\;\mathrm{odds}=\frac{P(\mathrm{MS}\;\mathrm{activity}|\mathrm{sNfL}\;\mathrm{result})}{1-P(\mathrm{MS}\;\mathrm{activity}|\mathrm{sNfL}\;\mathrm{result})}$$

Bayesian updating can then be expressed as

Post-test odds = Pre-test odds × Likelihood ratio

### 4.4. Likelihood Ratio and Test Characteristics

For a dichotomised biomarker interpretation (e.g., elevated vs. non-elevated sNfL), the positive and negative likelihood ratios (*LR*^+^ and *LR*^−^) can be calculated from the test characteristics, namely sensitivity and specificity:

$$LR^{+}=\frac{\mathrm{Sensitivity}}{1-\mathrm{Specificity}},\;\;\;\;\;\;\;\;LR^{-}=\frac{1-\mathrm{Sensitivity}}{\mathrm{Specificity}}$$

where

- **Sensitivity** is the probability that sNfL is elevated in patients with true inflammatory disease activity.
- **Specificity** is the probability that sNfL is not elevated in patients without inflammatory disease activity.

The *LR*^+^ quantifies how strongly an elevated sNfL result shifts the probability toward disease activity, whereas the *LR*^−^ quantifies how strongly a non-elevated result shifts the probability away from it.

### 4.5. Back-Transformation to Probability

To convert post-test odds back into a clinically interpretable probability, the following relationship is used:

$$P(\mathrm{MS}\;\mathrm{activity}|\mathrm{sNfL}\;\mathrm{result})=\frac{\mathrm{Post}\text{-}\mathrm{test}\;\mathrm{odds}}{1+\mathrm{Post}\text{-}\mathrm{test}\;\mathrm{odds}}$$

This allows clinicians to translate Bayesian updating into absolute risk estimates.

### 4.6. Clinical Example

Assume the following illustrative scenario, based on reported diagnostic performance estimates for sNfL in MS [50]:

- Pre-test probability of inflammatory MS activity (e.g., in the context of a clinically suspected relapse) = 30%
- Sensitivity of elevated sNfL = 0.81
- Specificity = 0.70

First, calculate the positive likelihood ratio:

$$LR^{+}=\frac{0.81}{1-0.70}=\frac{0.81}{0.30}=2.7$$

Next, convert pre-test probability into odds:

$$\mathrm{Pre}\text{-}\mathrm{test}\;\mathrm{odds}=\frac{0.30}{0.70}\approx 0.43$$

Update using Bayes’ rule in odds form:

Post-test odds = 0.43×2.7≈1.16

Finally, convert back to probability:

$$P(\mathrm{MS}\;\mathrm{activity}|\mathrm{elevated}\;\mathrm{sNfL})=\frac{1.16}{1+1.16}\approx 0.54$$

Thus, in this illustrative scenario, an elevated sNfL value would increase the estimated probability of inflammatory disease activity from 30% to approximately 54%.

## 5. Context-Dependent Uncertainty in sNfL Interpretation

Further clinical scenarios are exemplified in Table 1, illustrating that the informative value of sNfL is greatest at intermediate pre-test probabilities—such as in the presence of clinical suspicion of disease activity or new MRI lesions—than in both clinically stable and clearly active disease. At low pre-test probability, an isolated elevation of sNfL levels may lead to an overestimation of disease activity. Thus, as outlined above, sNfL should not be conceptualised as a binary marker of disease activity but rather as a probabilistic modifier within a Bayesian decision framework. Its clinical relevance arises—at least in theory—from integration into the overall clinical context, including clinical findings, MRI, and treatment history.

In practice, the clinical situation in patients with MS is frequently characterised by non-specific symptoms, heterogeneous disease courses, and variable treatment effects. In addition, the overall clinical picture may be cumulatively confounded by comorbidities. Accordingly, the sensitivity and specificity of sNfL for detecting MS disease activity are not universally fixed properties but depend on the clinical context and patient population [50,51,52,53,54]. Moreover, the individual pre-test probability of subclinical disease activity is often only crudely estimable and constitutes a key reason for clinicians to seek additional evidence. This implies that the parameters underlying the Bayesian estimation of post-test probability are subject to substantial variability—a classical problem of uncertainty propagation and, in epistemological terms, of error propagation under parameter uncertainty [7,55,56,57]. This cumulative uncertainty limits the reliability of interpreting single biomarker measurements in routine clinical care.

In addition, spectrum bias must be considered. The comparison of clearly active vs. stable clinical courses in validation studies may overestimate apparent discriminative performance and limit generalisability to clinically heterogeneous populations [50,58,59,60]. In real-world clinical practice, patients frequently present with minimal or uncertain disease activity. Within this intermediate clinical spectrum, the discriminative performance of the biomarker may be lower than in study populations with extreme contrasts, as test performance metrics are substantially influenced by disease severity, pre-test probability, comorbidities, and underlying case mix [61]. 

There is also a risk of partial verification bias, as not every elevation of sNfL levels in MS is systematically verified by MRI or clinical follow-up.

In summary, the use of a biologically plausible but context-dependent biomarker in a clinically uncertain setting constitutes a structural problem of iterative uncertainty propagation. An uncertain pre-test probability (individual disease activity) meets uncertain test parameters (context-dependent sensitivity and specificity and the absence of universally standardised cut-offs). Scientifically, this represents a problem of misclassification under parameter uncertainty, amplified by spectrum bias, base-rate dependence, and decision noise. Valid use of sNfL therefore requires longitudinal assessment, age-adjusted reference ranges, careful consideration of confounding factors such as BMI and comorbidities, and integration into multimodal decision models rather than interpretation of isolated values.

At present, however, a relevant evidence gap remains, as there is a lack of prospective multicentre studies systematically evaluating the role of sNfL in individualised treatment management of patients with MS in routine clinical care [9].

## 6. Outlook for Multimodal Biomarker Integration

Building on the framework for interpreting sNfL results discussed above, which integrates clinical and MRI-based findings with a molecular biomarker of neuroaxonal injury, Bayesian reasoning can, in principle, be extended from single-biomarker to multimarker interpretation. Future decision frameworks may incorporate additional molecular biomarkers, provided that their joint diagnostic information is validated rather than assuming that individual biomarker effects are independent.

Depending on the clinical context, further markers of tissue injury could become particularly relevant. These include GFAP as an indicator of astrocytic injury and SNAP25 as a marker of synaptic neuronal injury and degenerative processes associated with disease progression. Biomarkers reflecting immune-cell activation, such as BCMA for B-cell activation, sCD27 for T-cell activation, CHI3L1 and OPN as markers of microglial activation, and cytokines such as IL-6 and IL-17, also represent promising candidates [62,63].

However, their value for routine clinical assessment of MS remains limited. For markers of tissue destruction, this mainly reflects the lack of validation studies demonstrating added value for real-world clinical care. For immunological markers, clinical implementation is particularly constrained by substantial biological, pre-analytical, and methodological variability.

## Figures and Tables

**Table 1:** Context-dependent interpretation of serum neurofilament light chain results in multiple sclerosis.

Pre-Test Probability Zone	Typical Clinical Context	sNfL Result	Expected Post-Test Implication	Clinical Usefulness of the Test	Interpretation with Confounders ^1^
Low (<20%)	Clinically stable patient; MRI stable	Normal	Probability of disease activity becomes very low	Moderate—supports reassurance and conservative management	Interpretation remains robust
		Elevated	Probability increases moderately; possible subclinical activity	Moderate—prompts closer monitoring or repeat imaging	Reduced specificity; alternative explanations should be considered
Intermediate (20–60%)	Uncertain clinical situation; equivocal MRI findings	Normal	Probability decreases substantially; disease activity less likely	High—may prevent unnecessary treatment escalation	Interpretation largely preserved; clinical context remains essential
		Elevated	Probability increases substantially; disease activity more likely	Very high—may prompt treatment escalation or intensified surveillance	Reduced specificity; alternative causes possible; interpret with caution
High (>60%)	Evident clinical worsening; new MRI lesions	Normal	Probability decreases somewhat but remains significant; uncertainty persists	Moderate—may trigger search for alternative explanations	Potential for false reassurance; consider confounders and timing
		Elevated	Probability becomes very high	Confirmatory—increases confidence rather than changing management	Limited additional impact; confounders unlikely to change interpretation

^1^ While age and body mass index can be partially accounted for (e.g., using z-scores), sNfL levels may also be influenced by comorbid conditions (e.g., infection, trauma, vascular, or other neurological and non-neurological disorders), which can reduce specificity for disease activity.

## Data Availability

No new data were created for this article.

## Abbreviations

BCMA	B-cell maturation antigen
BMI	body mass index
CD	cluster of differentiation
CHI3L1	chitinase-3-like protein 1
CSF	cerebrospinal fluid
ECLIA	electrochemiluminescence immunoassay
EDSS	Expanded Disability Status Scale
ELISA	enzyme-linked immunosorbent assay
GFAP	glial fibrillary acidic protein
IL	interleukin
LR	likelihood ratio
MRI	magnetic resonance imaging
MS	multiple sclerosis
NEDA	no evidence of disease activity
NfL	neurofilament light chain
OPN	osteopontin
PIRA	progression independent of relapse activity
PIRMA	progression independent of relapse and MRI activity
sCD27	soluble CD27
Simoa	single-molecule array
SNAP25	synaptosomal-associated protein 25
sNfL	serum neurofilament light chain
